# Supplementary material for: Quantitative assessment of multiple pathogen exposure and immune dynamics at scale
Source: Microbiol Spectr. 2023 Dec 8;12(1):e02399-23. doi: 10.1128/spectrum.02399-23 (PMC10783028; doi:10.1128/spectrum.02399-23)
Supplement: Supplemental material — Fig. S1 to S8; Tables S1 to S4. [file spectrum.02399-23-s0001.docx]

Supplementary Materials for

**Quantitative assessment of multiple pathogen exposure and immune dynamics at scale**

Lusheng Song *et al.*

*Corresponding author. Email: jlabaer@asu.edu

**This file includes:**

Figs. S1 to S8

Tables S1 to S4


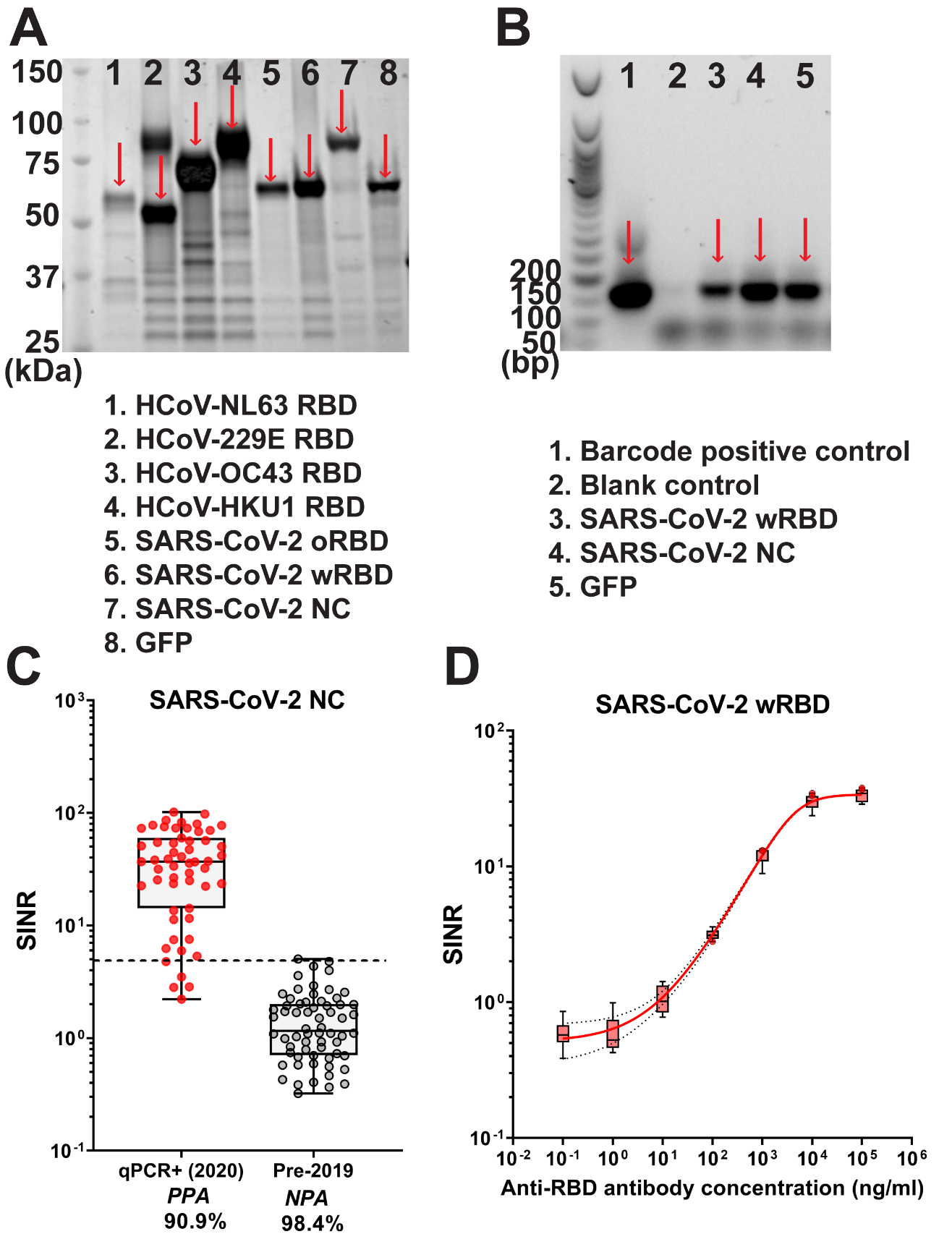


Fig. S1.

Three protein MISPA library qualification.

(A). In-gel fluorescence assay result of receptor-binding domain (RBD) proteins from seasonal coronavirus and SARS-CoV-2. All RBD proteins were expressed using the Expi293F system, and SARS-CoV-2 NC and GFP were expressed in IVTT. All proteins were produced with a C terminal HaloTag that attaches covalently to fluorescently-labeled chloroalkane ligand. The red arrows indicate expressed proteins of the expected size.

(B). Protein barcoding quality control DNA agarose gel image. The bands at 156 bp indicated proper barcoding compared to the blank control.

(C). Clinical agreement for nucleocapsid (NC).

(D). Limit of detection (LOD) for RBD of SARS-CoV-2 Wuhan strain (wRBD) MISPA. A 5-parameter logistic regression model fitted the MISPA response. The LOD was determined using cutoffs at five times blank sample (1% BSA).

SINR, Spike-In-Normalized Ratio.


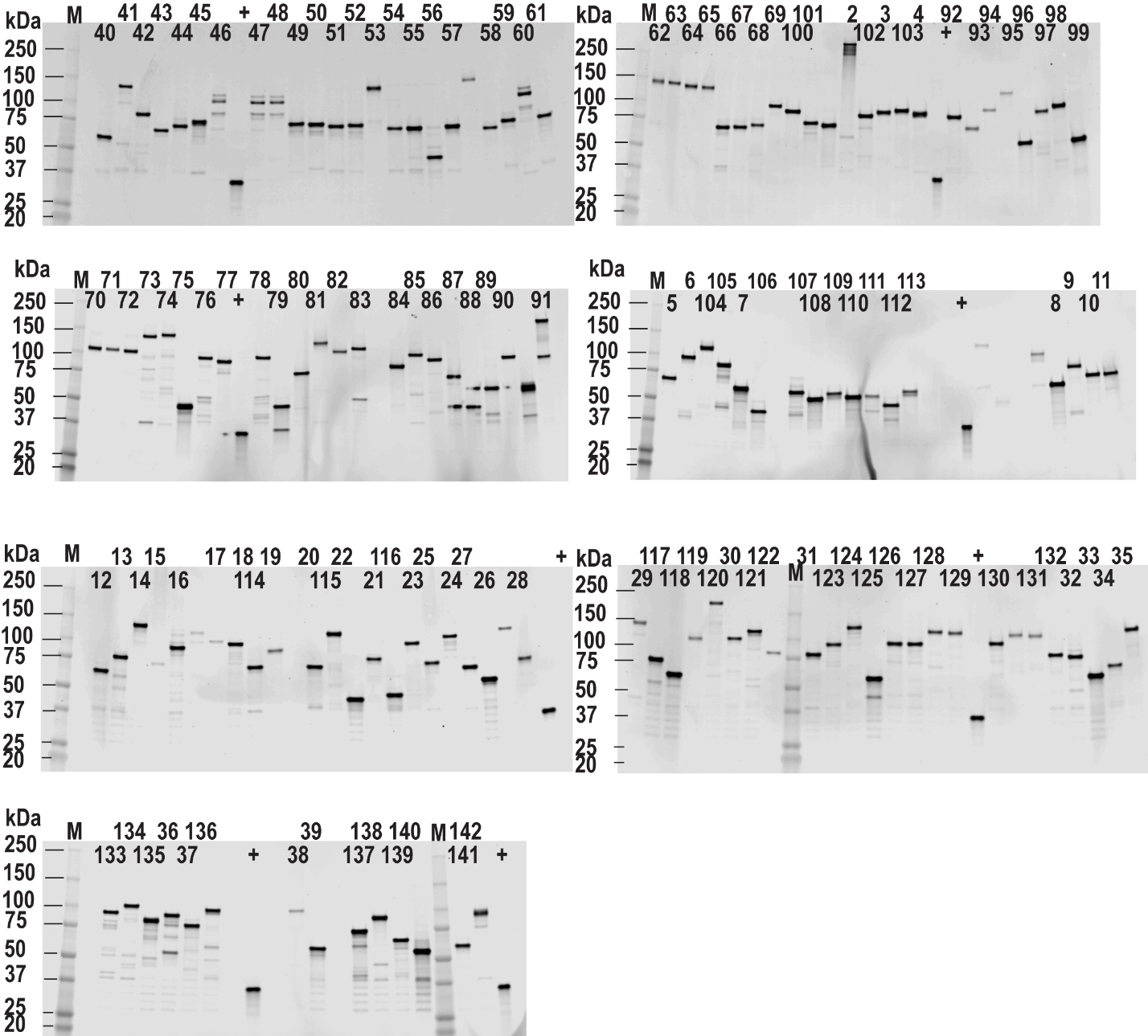


Fig. S2.

Protein expression qualification.

In-gel fluorescence images of 142 antigens expressed in IVTT that were used in MISPA analysis. The number for each protein is the same as in Supplementary Table 1. +, purified HaloTag protein; M, marker lane.


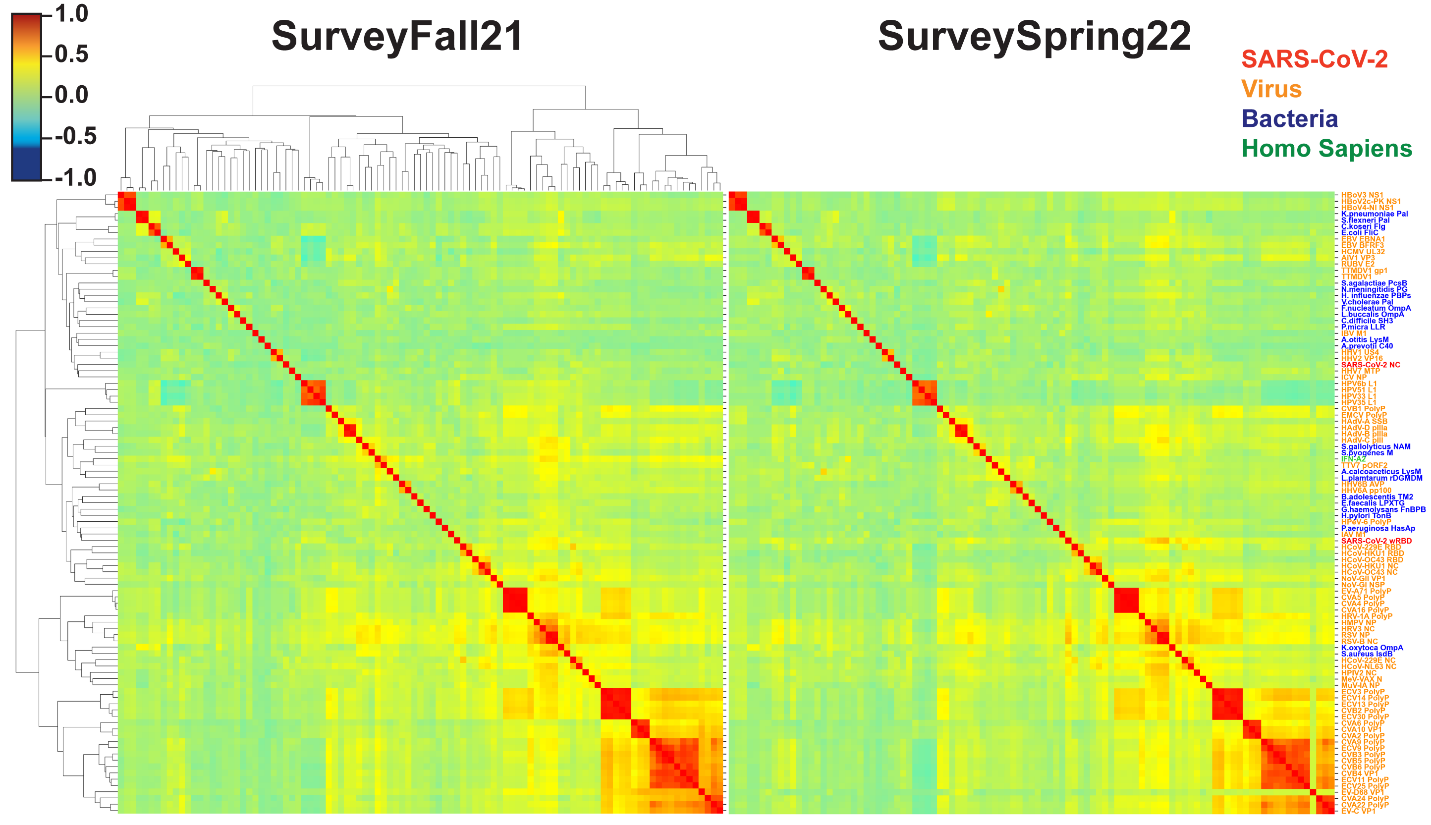


Fig. S3.

A set of 99 antigens (>5% sero-prevalence) were clustered by the pairwise Pearson correlation of the responses in SurveyFall21 (left). The Pearson correlation (R) values were indicated in color schemes. The same antigen order was applied to the responses in SurveySpring22 (right) for comparison. When the SurveySpring22 data were independently clustered, the Rand index (i.e., cluster similarity) between SurveyFall21 and SurveySpring22 was 0.859 (*p*<0.001).


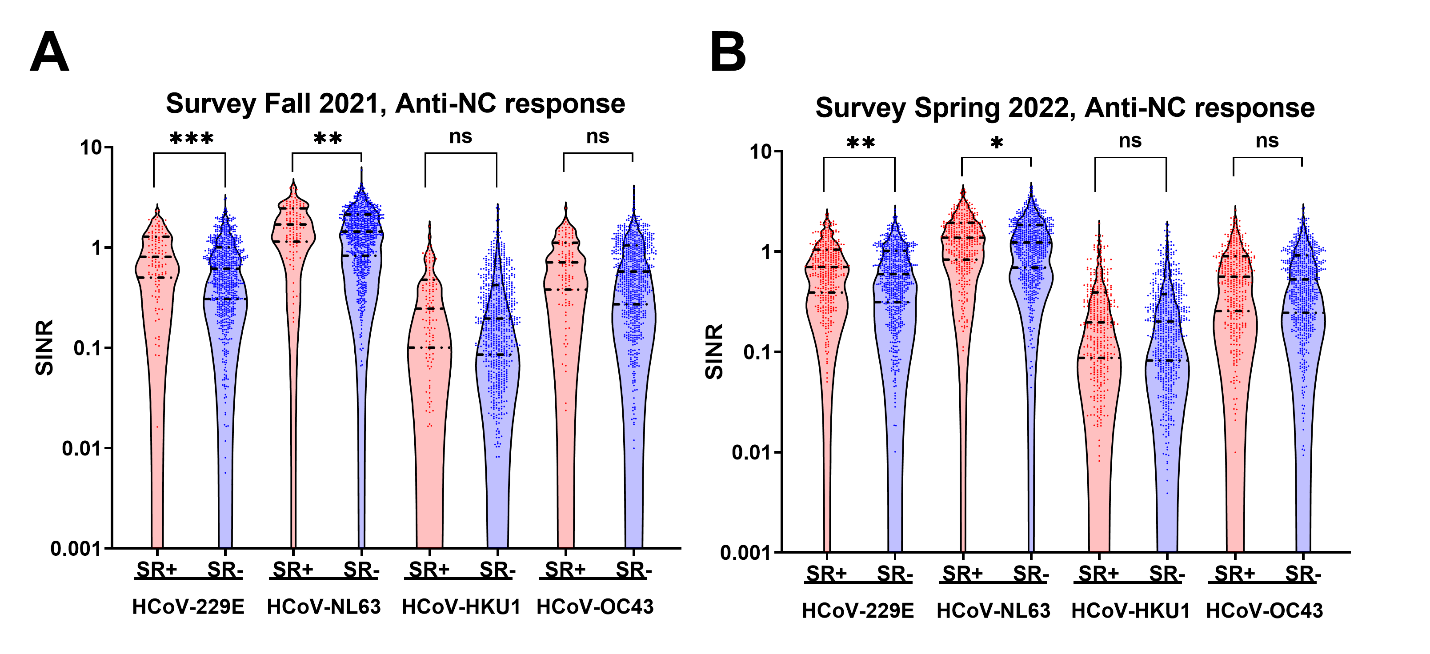


Fig. S4.

Antibody levels difference against NC proteins from coronaviruses (alpha coronavirus (HCoV-229E, HCoV-NL63), beta coronavirus (HCoV-HKU1, HCoV-OC43)) between SR+ and SR- group in both SurveyFall21 (A) and SurveySpring22 (B). SR+ and SR- indicate self-reported COVID-19 positive and negative, both confirmed with Bio-Rad Platelia SARS-CoV-2 total Ab ELISA assay. The median, 25^th^ and 75^th^ percentiles were shown as dotted lines. RankSum test p value was indicated on top of each antigen. ***, p<0.001; **, p<0.01; *, p<0.05; ns, not significant.

**
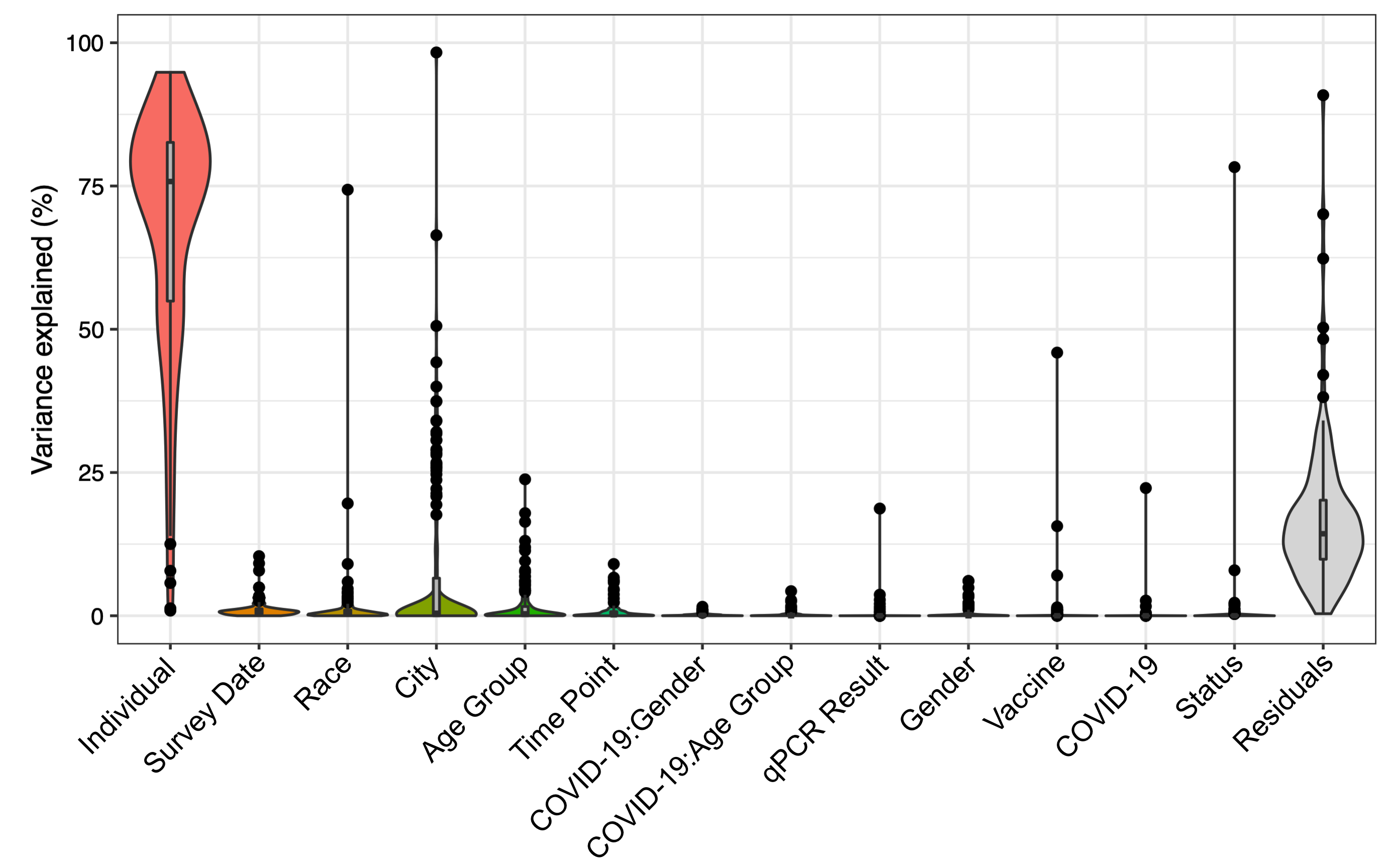
**

Fig. S5.

Variance Partition analysis to model proportions of variability in antibody responses that are explained by known covariates. Antibody abundance data from both SurveyFall21 and SurveySpring22 included in analysis.


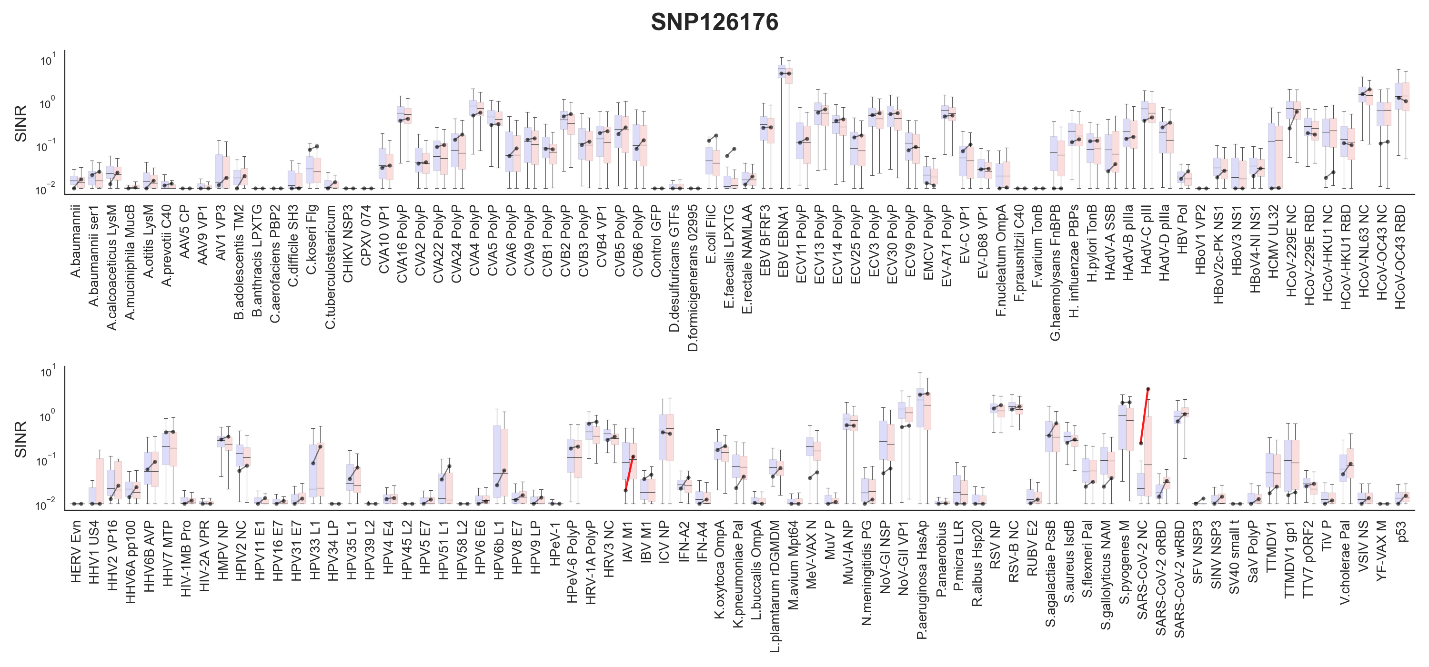


Fig. S6.

Antibody response levels for Participant SNP126176 (black dots) for antibodies to all 147 antigens for both surveys (SurveyFall21; SurveySpring22). These are shown in the context of the population responses for all 137 participants who were assessed in both surveys (box indicates median and 25th – 75th percentile, whiskers max and min samples). Changes in the participant’s antibody levels between the two time points are indicated by red/black lines to indicate more than or less than a 5-fold change, respectively. SINR, Spike-In-Normalized Ratio.


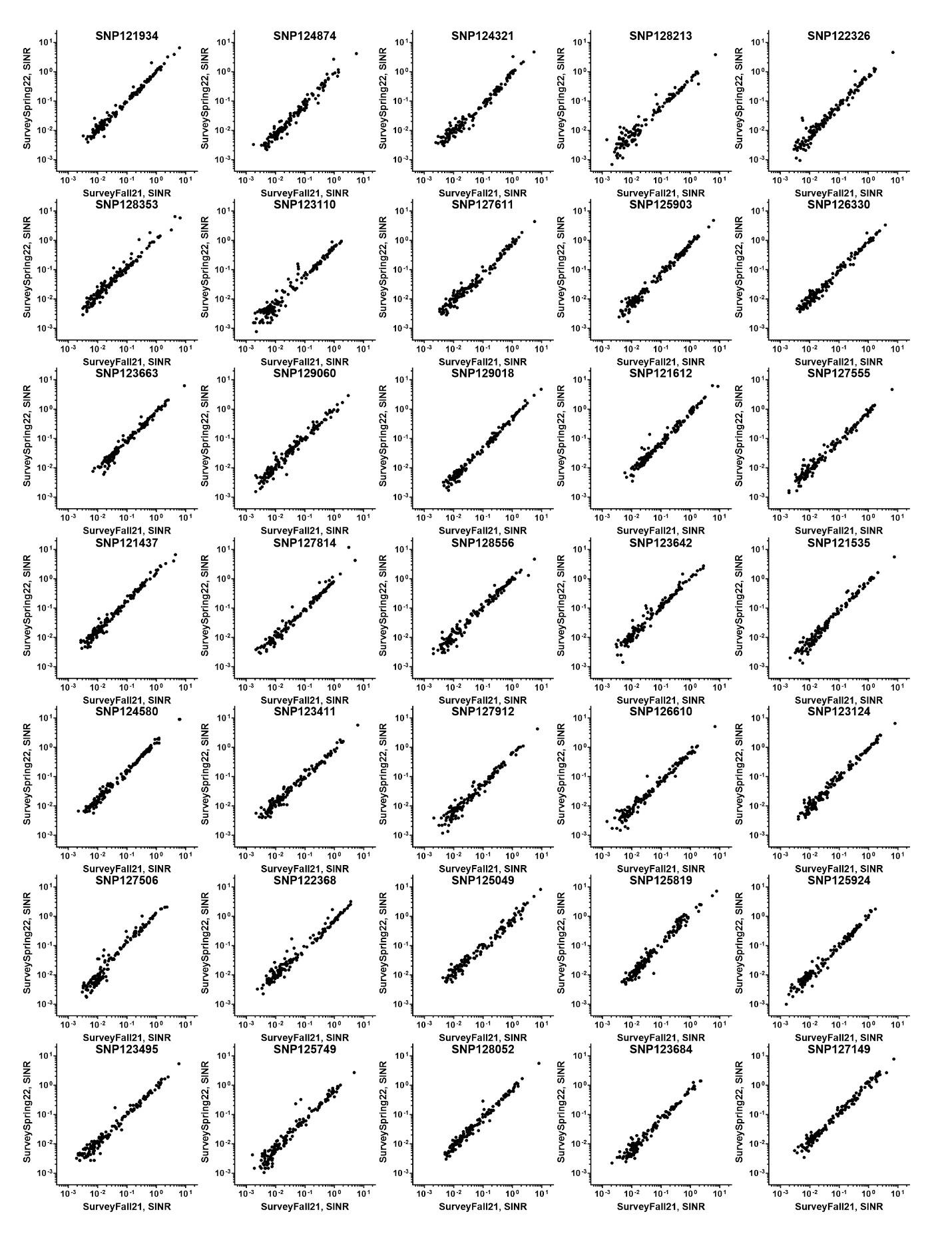

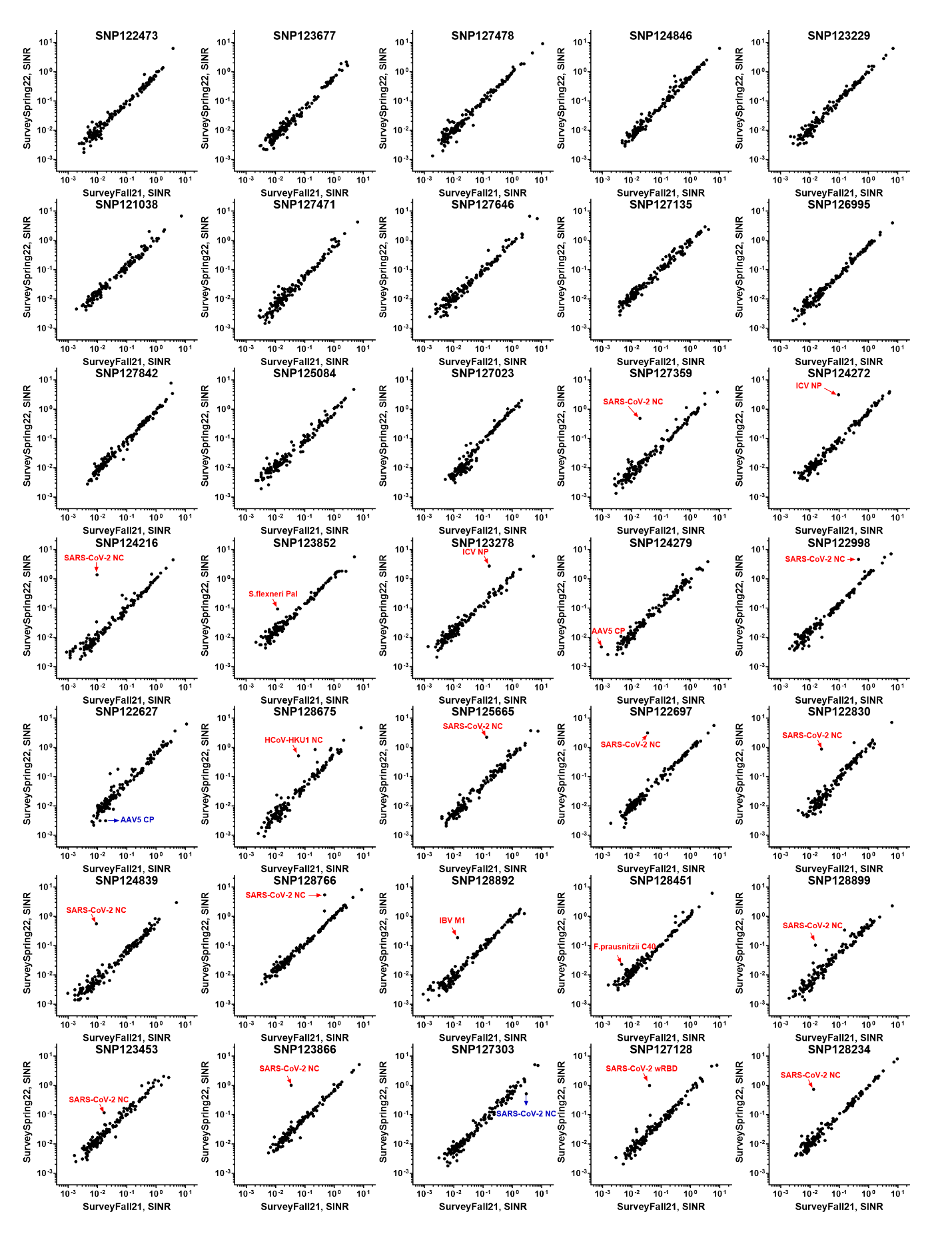

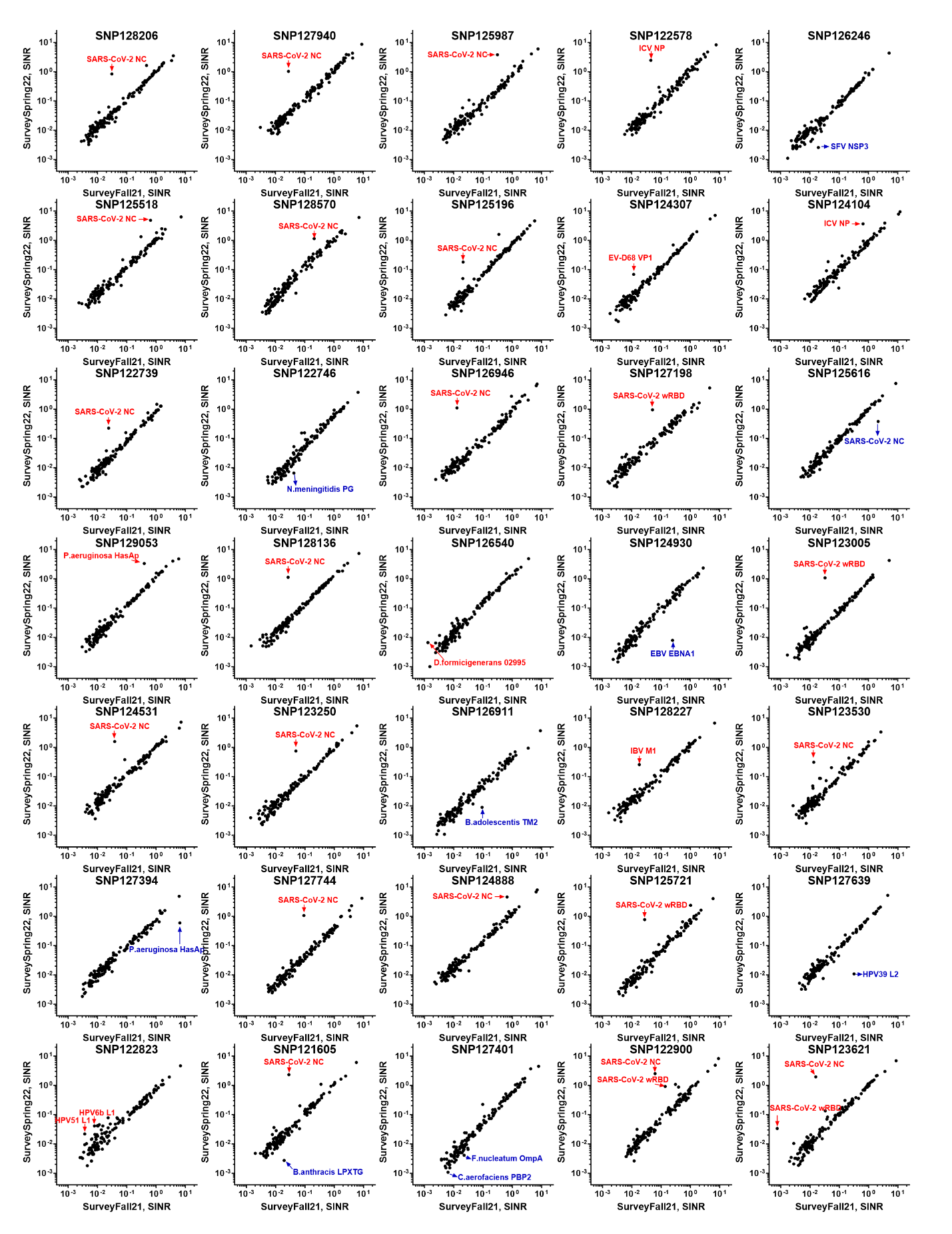

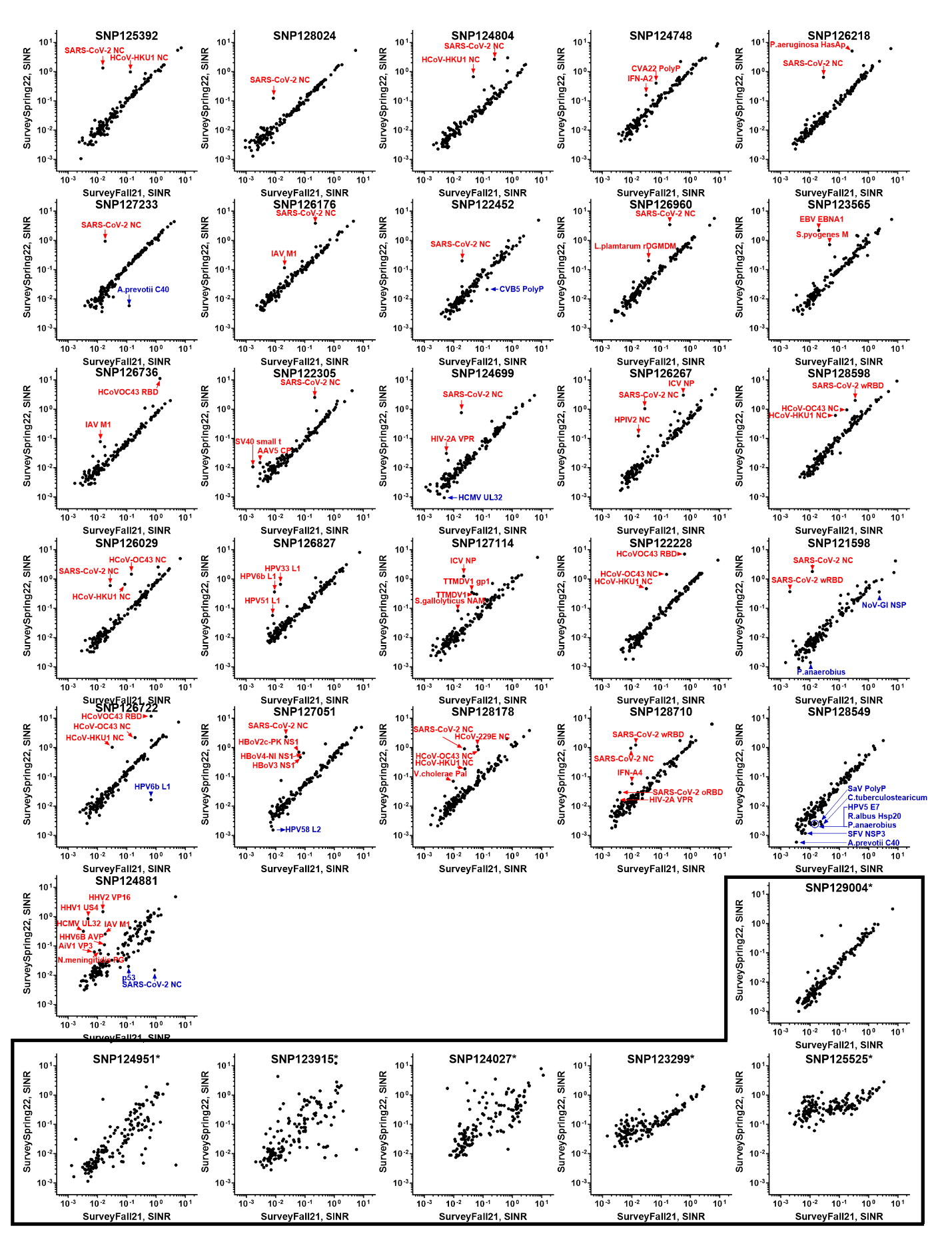


**Fig. S7.**

Longitudinal comparison of seroreactivity against 147 antigens between the two serosurveys for all 137 participants. For the 137 participants who were assessed in both serosurveys, the antibody response levels to all 147 antigens are self-compared between SurveyFall21 and SurveySpring22. Individuals with more than or equal to 5-fold antibodies changes were highlighted with red arrows and the antigens are indicated. There were six samples (*) that had abnormal changes due to technical issues. SINR, Spike-In-Normalized Ratio.


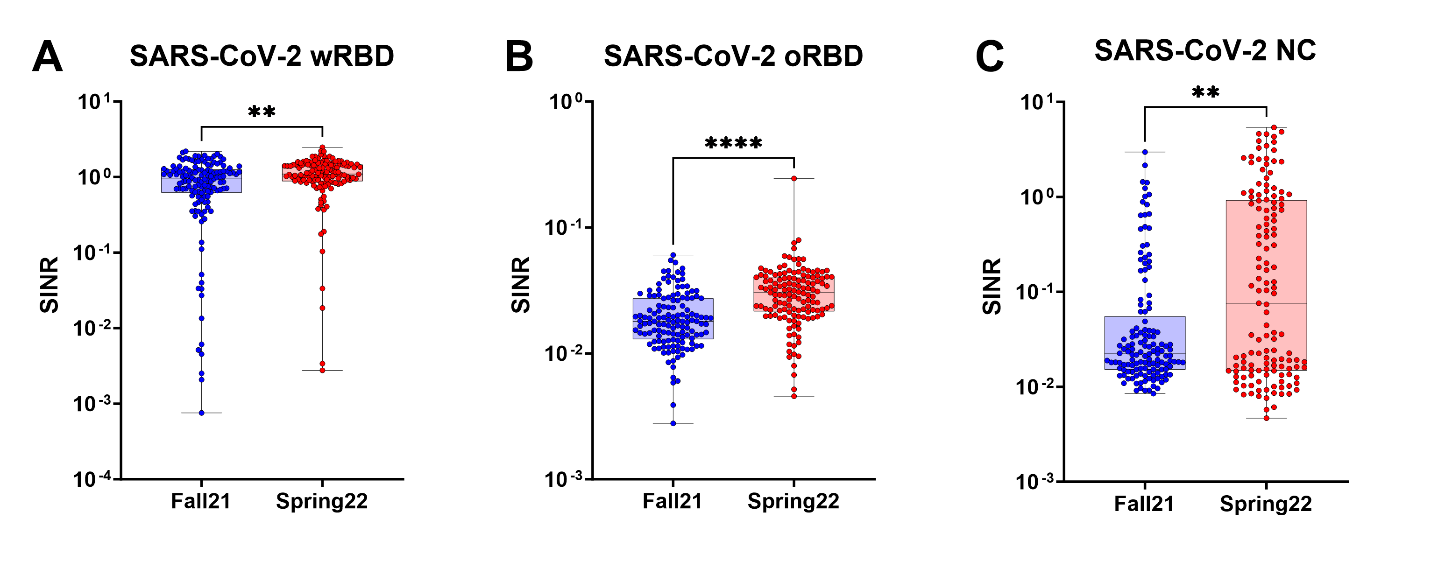


Fig. S8.

Antibody response to SARS-CoV-2 antigens for 137 individuals that participated in both surveys (SurveyFall21; SurveySpring22). The anti-SARS-CoV-2 wRBD (A), oRBD (B), and NC (C) for all 137 participants who were assessed in both surveys (box indicates median and 25th – 75th percentile, whiskers max and min samples). RankSum test p value was indicated on top of each antigen. ****, p<0.0001; **, p<0.01.

Table S1.

Antigen list for MISPA library. The antigen name is based on the species/strain and protein names. The group of bacteria, viruses, and homo sapiens are listed. If available, the species/strain and taxon ID use the NCBI standard name. The protein name is the full name of each antigen. The expression method is listed for each antigen. The protein index-1 to index-39 are bacteria proteins, proteins from index-40 to index-138, and from index-143 to index-147 are virus proteins, proteins with index-140, 141, 142 are human proteins, and index-139 protein is GFP. All proteins were expressed in IVTT except for the RBD proteins, which were expressed in Expi293F cells.

| Index | Antigen Name | Species/Strain | Taxon ID | Protein Name |
| --- | --- | --- | --- | --- |
| 1 | *M.avium* Mpt64 | *Mycobacterium avium subsp. paratuberculosis* | 1770 | Immunogenic protein MPT64 |
| 2 | *S.aureus* IsdB | *Staphylococcus aureus subsp. aureus COL* | 93062 | Iron-regulated surface determinant protein B, IsdB |
| 3 | *S.agalactiae* PcsB | *Streptococcus agalactiae 2603V/R* | 208435 | PcsB protein |
| 4 | *S.flexneri* Pal | *Shigella flexneri 2a* | 42897 | peptidoglycan-associated lipoprotein Pal |
| 5 | *S.gallolyticus* NAM | *Streptococcus gallolyticus subsp. gallolyticus ATCC 43143* | 981539 | N-acetylmuramidase |
| 6 | *S.pyogenes* M | *Streptococcus pyogenes M1 GAS* | 160490 | M protein |
| 7 | *V.cholerae* Pal | *Vibrio cholerae O1 biovar El Tor str. N16961* | 243277 | peptidoglycan-associated lipoprotein |
| 8 | *P.aeruginosa* HasAp | *Pseudomonas aeruginosa* | 287 | heme acquisition protein HasAp |
| 9 | *A.baumannii* | *Acinetobacter baumannii ATCC 17978* | 400667 | hypothetical protein |
| 10 | *A.baumannii* ser1 | *Acinetobacter baumannii* | 470 | sel1 repeat family protein |
| 11 | *A.calcoaceticus* LysM | *Acinetobacter calcoaceticus* | 471 | LysM peptidoglycan-binding domain-containing protein |
| 12 | *A.muciniphila* MucB | *Akkermansia muciniphila ATCC BAA-835* | 349741 | sigma E regulatory protein MucB/RseB |
| 13 | *A.otitis* LysM | *Alloiococcus otitis ATCC 51267* | 883081 | LysM peptidoglycan-binding domain-containing protein |
| 14 | *A.prevotii* C40 | *Anaerococcus prevotii DSM 20548* | 525919 | NlpC/P60 family protein |
| 15 | *B.anthracis* LPXTG | *Bacillus anthracis str. Ames* | 198094 | Putative LPXTG-motif cell wall anchor domain protein |
| 16 | *B.adolescentis* TM2 | *Bifidobacterium adolescentis ATCC 15703* | 367928 | TM2 domain-containing protein |
| 17 | *C.aerofaciens* PBP2 | *Collinsella aerofaciens ATCC 25986* | 411903 | penicillin-binding protein 2 |
| 18 | *C.difficile* SH3 | *Clostridioides difficile 630* | 272563 | ligand-binding protein SH3 |
| 19 | *C.koseri* Flg | *Citrobacter koseri ATCC BAA-895* | 290338 | flagellin |
| 20 | *C.tuberculostearicum* | *Corynebacterium tuberculostearicum SK141* | 553206 | Not available |
| 21 | *D.desulfuricans* GTFs | *Oleidesulfovibrio alaskensis G20* | 207559 | glycosyltransferase family 4 protein |
| 22 | *D.formicigenerans* 02995 | *Dorea formicigenerans ATCC 27755* | 411461 | hypothetical protein DORFOR_02995 |
| 23 | *E.coli* FliC | *Escherichia coli str. K-12 substr. MG1655* | 511145 | H48 family flagellin FliC |
| 24 | *E.faecalis* LPXTG | *Enterococcus faecalis V583* | 226185 | LPXTG cell wall anchor domain-containing protein |
| 25 | *E.rectale* NAMLAA | *[Eubacterium] rectale DSM 17629* | 657318 | N-acetylmuramoyl-L-alanine amidase |
| 26 | *F.nucleatum* OmpA | *Fusobacterium nucleatum subsp. nucleatum ATCC 25586* | 190304 | OmpA family protein |
| 27 | *F.prausnitzii* C40 | *Faecalibacterium prausnitzii A2-165* | 411483 | C40 family peptidase |
| 28 | *F.varium* TonB | *Fusobacterium varium ATCC 27725* | 469618 | TonB family domain protein |
| 29 | *G.haemolysans* FnBPB | *Gemella haemolysans ATCC 10379* | 546270 | fibronectin-binding SSURE repeat-containing protein |
| 30 | *H. influenzae* PBPs | *Haemophilus influenzae Rd KW20* | 71421 | penicillin-binding protein activator |
| 31 | *H.pylori* TonB | *Helicobacter pylori 26695* | 85962 | energy transducer TonB |
| 32 | *K.oxytoca* OmpA | *Klebsiella oxytoca (Flugge 1886) Lautrop 1956* | 571 | Outer membrane protein A |
| 33 | *K.pneumoniae* Pal | *Klebsiella pneumoniae subsp. pneumoniae MGH 78578* | 272620 | peptidoglycan-associated lipoprotein Pal |
| 34 | *L.buccalis* OmpA | *Leptotrichia buccalis C-1013-b* | 523794 | OmpA/MotB domain protein |
| 35 | *L.plamtarum* rDGMDM | *Lactiplantibacillus plantarum WCFS1* | 220668 | gamma-D-glutamate-meso-diaminopimelate muropeptidase |
| 36 | *N.meningitidis* PG | *Neisseria meningitidis Z2491* | 122587 | peptidoglycan DD-metalloendopeptidase family protein |
| 37 | *P.anaerobius* | *Peptostreptococcus anaerobius VPI 4330 = DSM 2949* | 1035196 | cell wall-binding repeat-containing protein |
| 38 | *P.micra* LLR | *Parvimonas micra ATCC 33270* | 411465 | leucine-rich repeat domain-containing protein |
| 39 | *R.albus* Hsp20 | *Ruminococcus albus 7 = DSM 20455* | 697329 | Hsp20/alpha crystallin family protein |
| 40 | AiV1 VP3 | Aichi virus 1 | 1313215 | Capsid protein VP3 |
| 41 | AAV5 CP | Adeno-associated virus 5 | 82300 | capsid protein |
| 42 | AAV9 VP1 | Adeno-associated virus 9 | 235455 | Capsid protein VP1 |
| 43 | HPV4 E4 | Betapapillomavirus 4 | 334208 | E4 protein |
| 44 | CVA9 PolyP | Coxsackievirus A9 | 12067 | polyprotein |
| 45 | CVA24 PolyP | Coxsackievirus A24 | 12089 | polyprotein |
| 46 | CVA5 PolyP | Coxsackievirus A5 | 42786 | polyprotein |
| 47 | CVA4 PolyP | Coxsackievirus A4 | 42785 | polyprotein |
| 48 | CVA16 PolyP | Coxsackievirus A16 | 31704 | polyprotein |
| 49 | CVA22 PolyP | Coxsackievirus A22 | 42783 | polyprotein |
| 50 | CVA6 PolyP | Coxsackievirus A6 | 86107 | polyprotein |
| 51 | CVA10 VP1 | Coxsackievirus A10 | 42769 | Capsid protein VP1 |
| 52 | CVA2 PolyP | Coxsackievirus A2 | 33757 | polyprotein |
| 53 | CVB2 PolyP | Coxsackievirus B2 | 82639 | polyprotein |
| 54 | CVB3 PolyP | Coxsackievirus B3 | 12072 | polyprotein |
| 55 | CVB5 PolyP | Coxsackievirus B5 | 12074 | polyprotein |
| 56 | CVB1 PolyP | Coxsackievirus B1 | 12071 | polyprotein |
| 57 | CVB6 PolyP | Coxsackievirus B6 | 74561 | polyprotein |
| 58 | CPXV 074 | Cowpox virus | 10243 | CPXV074 protein |
| 59 | ECV9 PolyP | Echovirus 9 strain Barty | 103914 | polyprotein |
| 60 | EV-A71 PolyP | Enterovirus A71 | 39054 | polyprotein |
| 61 | EV-D68 VP1 | Enterovirus D68 | 42789 | Capsid protein VP1 |
| 62 | ECV3 PolyP | Echovirus E3 | 47516 | polyprotein |
| 63 | ECV14 PolyP | Echovirus E14 | 47502 | polyprotein |
| 64 | ECV13 PolyP | Echovirus E13 | 47501 | polyprotein |
| 65 | ECV30 PolyP | Echovirus E30 | 41846 | polyprotein |
| 66 | ECV11 PolyP | Echovirus E11 | 12078 | polyprotein |
| 67 | ECV25 PolyP | Echovirus E25 | 45101 | polyprotein |
| 68 | EMCV PolyP | Encephalomyocarditis virus | 12104 | polyprotein |
| 69 | HHV2 VP16 | Human alphaherpesvirus 2 | 10310 | Tegument protein VP16 |
| 70 | HBoV2c-PK NS1 | Human bocavirus 2c PK | 1511882 | Initiator protein NS1 |
| 71 | HBoV3 NS1 | Human bocavirus 3 | 638313 | Initiator protein NS1 |
| 72 | HBoV4-NI NS1 | Human bocavirus 4 NI | 1511883 | Initiator protein NS1 |
| 73 | HHV6B AVP | Human betaherpesvirus 6B | 32604 | antigenic virion protein |
| 74 | HHV6A pp100 | Human betaherpesvirus 6A | 32603 | Pp100 |
| 75 | HHV7 MTP | Human betaherpesvirus 7 | 10372 | myristylated tegument protein |
| 76 | HAdV-D pIIIa | Human mastadenovirus D | 130310 | capsid protein precursor pIIIa |
| 77 | HAdV-A SSB | Human mastadenovirus A | 129875 | single-stranded DNA-binding protein |
| 78 | HAdV-B pIIIa | Human mastadenovirus B | 108098 | capsid protein precursor pIIIa |
| 79 | HIV-1MB Pro | HIV-1 M:B | 505185 | protease |
| 80 | RSV NP | Human orthopneumovirus | 11250 | nucleoprotein |
| 81 | HPeV-1 | Human parechovirus 1 | 12063 | hypothetical protein |
| 82 | HPV34 LP | Human papillomavirus type 34 | 333764 | late protein |
| 83 | HPV9 LP | Human papillomavirus 9 | 10621 | late protein |
| 84 | HPeV-6 PolyP | Human parechovirus 6 | 411152 | polyprotein |
| 85 | HPIV2 NC | Human orthorubulavirus 2 | 2560525 | nucleocapsid protein |
| 86 | HRV3 NC | Human respirovirus 3 | 11216 | nucleocapsid protein |
| 87 | HRV-1A PolyP | Rhinovirus A1 | 573824 | polyprotein |
| 88 | HIV-2A VPR | HIV-2 subtype A | 388908 | vpr protein |
| 89 | IBV M1 | Influenza B virus (B/Lee/1940) | 518987 | matrix protein M1 |
| 90 | ICV NP | Influenza C virus (C/Ann Arbor/1/50) | 11553 | nucleoprotein |
| 91 | NoV-GII VP1 | Norovirus GII | 122929 | Capsid protein VP1 |
| 92 | NoV-GI NSP | Norovirus GI/Hu/JP/2007/GI.P3_GI.3/Shimizu/KK2866 | 1529909 | nonstructural polyprotein |
| 93 | SaV PolyP | Salivirus NG-J1 | 651733 | polyprotein |
| 94 | TTMDV1 | Torque teno midi virus 1 | 687379 | hypothetical protein |
| 95 | TTV7 pORF2 | Torque teno virus 7 | 687346 | PORF2 |
| 96 | VSIV NS | Vesicular stomatitis Indiana virus | 11277 | Phosphoprotein NS |
| 97 | HCoV-HKU1 NC | Human coronavirus HKU1 | 290028 | nucleocapsid protein |
| 98 | HCoV-OC43 NC | Human coronavirus OC43 | 31631 | nucleocapsid protein |
| 99 | HCoV-229E NC | Human coronavirus 229E | 11137 | nucleocapsid protein |
| 100 | HCoV-NL63 NC | Human coronavirus NL63 | 277944 | nucleocapsid protein |
| 101 | RUBV E2 | Rubella virus strain M33 | 11043 | Spike glycoprotein E2 |
| 102 | TTMDV1 gp1 | Small anellovirus 1 | 289366 | Uncharacterized ORF3 protein |
| 103 | SFV NSP3 | Semliki Forest virus | 11033 | Non-structural protein 3 |
| 104 | SINV NSP3 | Sindbis virus | 11034 | Non-structural protein 3 |
| 105 | TiV P | Tioman virus | 162013 | Non-structural protein |
| 106 | YF-VAX M | Yellow fever virus | 11089 | Small envelope protein M |
| 107 | EBV BFRF3 | Human gammaherpesvirus 4 | 10376 | Small capsomere-interacting protein |
| 108 | HPV5 E7 | Human papillomavirus 5 | 333923 | E7 protein |
| 109 | HPV6 E6 | Human papillomavirus type 6 | 31552 | E6 protein |
| 110 | HPV8 E7 | Human papillomavirus type 8 | 10579 | E7 protein |
| 111 | HPV16 E7 | Human papillomavirus type 16 | 333760 | E7 protein |
| 112 | IAV M1 | Influenza A virus | 11320 | Matrix protein 1 |
| 113 | SV40 small t | Betapolyomavirus macacae | 1891767 | Small t antigen |
| 114 | CVB4 VP1 | Coxsackievirus B4 (strain E2) | 103905 | Capsid protein VP1 |
| 115 | CHIKV NSP3 | Chikungunya virus | 37124 | Non-structural protein 3 |
| 116 | EV-C VP1 | Enterovirus C | 138950 | Capsid protein VP1 |
| 117 | HHV1 US4 | Human alphaherpesvirus 1 | 10298 | Envelope glycoprotein G |
| 118 | HBV Pol | Hepatitis B virus | 10407 | polymerase |
| 119 | HERV Evn | Human Endogenous Retrovirus IDDMK1,2-22 | 64382 | Env polyprotein |
| 120 | HCMV UL32 | Human betaherpesvirus 5 | 10359 | Tegument protein pp150 |
| 121 | HAdV-C pIII | Human mastadenovirus C | 129951 | Penton protein pIII |
| 122 | HMPV NP | Human metapneumovirus | 162145 | Nucleoprotein |
| 123 | HPV6b L1 | Human papillomavirus type 6b | 10600 | Major capsid L1 protein |
| 124 | HPV11 E1 | Human papillomavirus 11 | 10580 | E1 protein |
| 125 | HPV31 E7 | Human papillomavirus 31 | 10585 | E7 protein |
| 126 | HPV33 L1 | Human papillomavirus 33 | 10586 | Major capsid L1 protein |
| 127 | HPV35 L1 | Human papillomavirus 35 | 10587 | Major capsid L1 protein |
| 128 | HPV39 L2 | Human papillomavirus 39 | 10588 | L2 protein |
| 129 | HPV45 L2 | Human papillomavirus 45 | 10593 | L2 protein |
| 130 | HPV51 L1 | Human papillomavirus 51 | 10595 | Major capsid L1 protein |
| 131 | HPV58 L2 | Human papillomavirus 58 | 10598 | L2 protein |
| 132 | RSV-B NC | Human respiratory syncytial virus B | 208895 | Nucleocapsid protein |
| 133 | MeV-VAX N | Measles morbillivirus | 11234 | Nucleoprotein |
| 134 | MuV-IA NP | Mumps orthorubulavirus | 2560602 | Nucleoprotein |
| 135 | MuV P | Mumps orthorubulavirus | 2560602 | Non-structural protein V |
| 136 | HBoV1 VP2 | Human bocavirus 1 | 689403 | Minor capsid protein VP1 |
| 137 | EBV EBNA1 | Human gammaherpesvirus 4 | 10376 | Epstein-Barr nuclear antigen 1 |
| 138 | SARS-CoV-2 NC | Severe acute respiratory syndrome coronavirus 2 | 2697049 | Nucleocapsid protein |
| 139 | Control GFP |  |  | green fluorescence protein |
| 140 | IFN-α2 | Homo sapiens | 9606 | Interferon regulatory factor alpha2 |
| 141 | IFN-α4 | Homo sapiens | 9606 | Interferon regulatory factor alpha4 |
| 142 | p53 | Homo sapiens | 9606 | Cellular tumor antigen p53 |
| 143 | SARS-CoV-2 wRBD | Severe acute respiratory syndrome coronavirus 2 | 2697049 | Receptor-binding domain, wuhan strain |
| 144 | SARS-CoV-2 oRBD | Severe acute respiratory syndrome coronavirus 2 | 2697049 | Receptor-binding domain, omicron strain |
| 145 | HCoV-229E RBD | Human coronavirus 229E | 11137 | Receptor-binding domain |
| 146 | HCoV-OC43 RBD | Human coronavirus OC43 | 31631 | Receptor-binding domain |
| 147 | HCoV-HKU1 RBD | Human coronavirus HKU1 | 290028 | Receptor-binding domain |

Table S2.

Serosurvey results of anti-RBD and anti-NC of September 2021 and March 2022.

|  | Serosurvey September 2021 (N=1060*) | | | |  | Serosurvey March 2022 (N=1379) | | | | |
| --- | --- | --- | --- | --- | --- | --- | --- | --- | --- | --- |
|  | **RBD** | | **NC** | |  | **RBD** | | **NC** | | |
|  | **Beckman** | **MISPA** | **BioRad** | **MISPA** |  | **Beckman** | **MISPA** | | **BioRad** | **MISPA** |
| Vax+ (978) | | | | | **Vax+ (1318)** | | | | | |
| COVID+ (182) | 179 (98.4%) | 182 (100.0%) | 103 (56.6%) | 153 (84.1%) | COVID+ (498) | 495 (99.4%) | 489 (98.2%) | | 355 (71.3%) | 434 (87.1%) |
| COVID- (796) | 725 (91.1%) | 785 (98.6%) | 73 (9.2%) | 154 (19.3%) | COVID- (820) | 795 (97.0%) | 813 (99.1%) | | 145 (17.7%) | 228 (27.8%) |
| Vax- (82) | | | | | **Vax- (61)** | | | | | |
| COVID+ (22) | 10 (45.5%) | 22 (100.0%) | 13 (59.1%) | 19 (86.4%) | COVID+ (30) | 21 (70.0%) | 28 (93.3%) | | 25 (83.3%) | 28 (93.3%) |
| COVID+ (60) | 21 (35.0%) | 32 (53.3%) | 20 (33.3%) | 30 (50.0%) | COVID+ (31) | 18 (58.1%) | 21 (67.7%) | | 12 (38.7%) | 17 (54.8%) |

*Not included in this analysis were 4 samples that did not have vaccination or COVID-19 information.

Table S3.

Combined analysis of unique subjects within SurveyFall21 and SurveySpring22 examining (A) overlap between individuals within lowest 5th percentile of antibody response indicates an unexpectedly large subpopulation of participants with low antibody response against both sCoV and SARS-CoV-2 NC (One-sided Fisher’s Exact Test), (B) who are also at increased likelihood for reporting to have never had COVID-19 (One-sided Fisher’s Exact Test).

| A |  |  |  |  |  |
| --- | --- | --- | --- | --- | --- |
|  | **Resistant set 1 (5pct)** | **Resistant set 2 (5pct)** | **Subjects overlapping** | **FDR** | **Fold change** |
|  | SARS-CoV-2 NC | hCoV-229E NC | 17 | 1.86E-04 | 2.89 |
|  | SARS-CoV-2 NC | hCoV-NL63 NC | 14 | 3.46E-03 | 2.38 |
|  | SARS-CoV-2 NC | hCoV-OC43 NC | 12 | 1.71E-02 | 2.04 |
|  | SARS-CoV-2 NC | hCoV-HKU1 NC | 11 | 3.05E-02 | 1.87 |
| B |  |  |  |  |  |
|  | **Resistant samples (5pct)** | **Self Reported COVID-19** | **Subjects overlapping** | **FDR** | **Fold change** |
|  | SARS-CoV-2 NC & hCoV-229E NC | No | 17 | 2.81E-02 | 1.37 |

Table S4.

Seroprevalence for 147 antigens in MISPA serosurvey on September 2021 and March 2022.

| Index | Antigen Name | Group | SurveyFall21 | SurveySpring22 | Seroprevalence mean +/- SD |
| --- | --- | --- | --- | --- | --- |
| 1 | *M.avium* Mpt64 | Bacteria | 9.2% | 8.2% | 8.7% +/- 0.7% |
| 2 | *S.aureus* IsdB | Bacteria | 99.9% | 100.0% | 100.0% +/- 0.1% |
| 3 | *S.agalactiae* PcsB | Bacteria | 93.1% | 92.6% | 92.9% +/- 0.4% |
| 4 | *S.flexneri* Pal | Bacteria | 85.2% | 74.8% | 80.0% +/- 7.4% |
| 5 | *S.gallolyticus* NAM | Bacteria | 87.8% | 81.7% | 84.8% +/- 4.3% |
| 6 | *S.pyogenes* M | Bacteria | 81.9% | 75.9% | 78.9% +/- 4.2% |
| 7 | *V.cholerae* Pal | Bacteria | 88.9% | 90.9% | 89.9% +/- 1.4% |
| 8 | *P.aeruginosa* HasAp | Bacteria | 94.9% | 98.2% | 96.5% +/- 2.3% |
| 9 | *A.baumannii* | Bacteria | 3.7% | 1.5% | 2.6% +/- 1.5% |
| 10 | *A.baumannii* ser1 | Bacteria | 24.9% | 33.8% | 29.3% +/- 6.3% |
| 11 | *A.calcoaceticus* LysM | Bacteria | 5.1% | 2.1% | 3.6% +/- 2.1% |
| 12 | *A.muciniphila* MucB | Bacteria | 3.7% | 1.6% | 2.6% +/- 1.5% |
| 13 | *A.otitis* LysM | Bacteria | 18.5% | 9.9% | 14.2% +/- 6.1% |
| 14 | *A.prevotii* C40 | Bacteria | 39.3% | 28.9% | 34.1% +/- 7.4% |
| 15 | *B.anthracis* LPXTG | Bacteria | 1.8% | 0.8% | 1.3% +/- 0.7% |
| 16 | *B.adolescentis* TM2 | Bacteria | 25.2% | 27.5% | 26.3% +/- 1.6% |
| 17 | *C.aerofaciens* PBP2 | Bacteria | 6.4% | 5.6% | 6.0% +/- 0.6% |
| 18 | *C.difficile* SH3 | Bacteria | 24.3% | 21.2% | 22.8% +/- 2.2% |
| 19 | *C.koseri* Flg | Bacteria | 84.7% | 83.0% | 83.8% +/- 1.2% |
| 20 | *C.tuberculostearicum* | Bacteria | 20.2% | 42.0% | 31.1% +/- 15.4% |
| 21 | *D.desulfuricans* GTFs | Bacteria | 1.7% | 0.9% | 1.3% +/- 0.5% |
| 22 | *D.formicigenerans* 02995 | Bacteria | 9.5% | 2.2% | 5.9% +/- 5.2% |
| 23 | *E.coli* FliC | Bacteria | 97.6% | 96.8% | 97.2% +/- 0.6% |
| 24 | *E.faecalis* LPXTG | Bacteria | 33.6% | 1.3% | 17.4% +/- 22.8% |
| 25 | *E.rectale* NAMLAA | Bacteria | 3.1% | 1.6% | 2.4% +/- 1.1% |
| 26 | *F.nucleatum* OmpA | Bacteria | 57.1% | 67.3% | 62.2% +/- 7.2% |
| 27 | *F.prausnitzii* C40 | Bacteria | 4.7% | 3.0% | 3.8% +/- 1.2% |
| 28 | *F.varium* TonB | Bacteria | 0.8% | 0.5% | 0.6% +/- 0.2% |
| 29 | *G.haemolysans* FnBPB | Bacteria | 89.2% | 82.0% | 85.6% +/- 5.1% |
| 30 | *H. influenzae* PBPs | Bacteria | 99.7% | 100.0% | 99.9% +/- 0.2% |
| 31 | *H.pylori* TonB | Bacteria | 63.0% | 49.1% | 56.1% +/- 9.8% |
| 32 | *K.oxytoca* OmpA | Bacteria | 94.6% | 89.3% | 92.0% +/- 3.7% |
| 33 | *K.pneumoniae* Pal | Bacteria | 90.2% | 77.1% | 83.6% +/- 9.3% |
| 34 | *L.buccalis* OmpA | Bacteria | 41.3% | 16.0% | 28.6% +/- 17.9% |
| 35 | *L.plamtarum* rDGMDM | Bacteria | 11.7% | 8.6% | 10.1% +/- 2.2% |
| 36 | *N.meningitidis* PG | Bacteria | 57.2% | 59.0% | 58.1% +/- 1.3% |
| 37 | *P.anaerobius* | Bacteria | 0.7% | 0.4% | 0.5% +/- 0.2% |
| 38 | *P.micra* LLR | Bacteria | 66.3% | 54.0% | 60.2% +/- 8.7% |
| 39 | *R.albus* Hsp20 | Bacteria | 20.3% | 22.4% | 21.3% +/- 1.5% |
| 40 | AiV1 VP3 | Virus | 28.7% | 34.7% | 31.7% +/- 4.2% |
| 41 | AAV5 CP | Virus | 9.6% | 4.8% | 7.2% +/- 3.4% |
| 42 | AAV9 VP1 | Virus | 2.0% | 1.9% | 1.9% +/- 0.1% |
| 43 | HPV4 E4 | Virus | 7.7% | 6.2% | 7.0% +/- 1.1% |
| 44 | CVA9 PolyP | Virus | 70.7% | 63.7% | 67.2% +/- 4.9% |
| 45 | CVA24 PolyP | Virus | 75.9% | 72.7% | 74.3% +/- 2.3% |
| 46 | CVA5 PolyP | Virus | 100.0% | 99.9% | 99.9% +/- 0.1% |
| 47 | CVA4 PolyP | Virus | 100.0% | 99.8% | 99.9% +/- 0.2% |
| 48 | CVA16 PolyP | Virus | 99.8% | 99.7% | 99.8% +/- 0.1% |
| 49 | CVA22 PolyP | Virus | 77.0% | 66.9% | 71.9% +/- 7.2% |
| 50 | CVA6 PolyP | Virus | 82.5% | 75.5% | 79.0% +/- 4.9% |
| 51 | CVA10 VP1 | Virus | 55.8% | 48.9% | 52.3% +/- 4.9% |
| 52 | CVA2 PolyP | Virus | 69.1% | 60.2% | 64.6% +/- 6.3% |
| 53 | CVB2 PolyP | Virus | 100.0% | 99.8% | 99.9% +/- 0.2% |
| 54 | CVB3 PolyP | Virus | 91.2% | 91.4% | 91.3% +/- 0.2% |
| 55 | CVB5 PolyP | Virus | 89.7% | 89.7% | 89.7% +/- 0.0% |
| 56 | CVB1 PolyP | Virus | 91.9% | 85.4% | 88.6% +/- 4.6% |
| 57 | CVB6 PolyP | Virus | 84.5% | 85.0% | 84.8% +/- 0.3% |
| 58 | CPXV 074 | Virus | 2.7% | 1.2% | 2.0% +/- 1.1% |
| 59 | ECV9 PolyP | Virus | 75.8% | 64.0% | 69.9% +/- 8.3% |
| 60 | EV-A71 PolyP | Virus | 99.9% | 99.6% | 99.8% +/- 0.2% |
| 61 | EV-D68 VP1 | Virus | 59.2% | 31.6% | 45.4% +/- 19.5% |
| 62 | ECV3 PolyP | Virus | 100.0% | 99.8% | 99.9% +/- 0.2% |
| 63 | ECV14 PolyP | Virus | 99.9% | 99.9% | 99.9% +/- 0.0% |
| 64 | ECV13 PolyP | Virus | 99.9% | 99.9% | 99.9% +/- 0.0% |
| 65 | ECV30 PolyP | Virus | 100.0% | 99.9% | 100.0% +/- 0.1% |
| 66 | ECV11 PolyP | Virus | 77.7% | 77.9% | 77.8% +/- 0.1% |
| 67 | ECV25 PolyP | Virus | 77.0% | 73.0% | 75.0% +/- 2.8% |
| 68 | EMCV PolyP | Virus | 20.6% | 16.2% | 18.4% +/- 3.1% |
| 69 | HHV2 VP16 | Virus | 46.4% | 43.6% | 45.0% +/- 2.0% |
| 70 | HBoV2c-PK NS1 | Virus | 58.8% | 46.9% | 52.8% +/- 8.4% |
| 71 | HBoV3 NS1 | Virus | 54.9% | 47.8% | 51.3% +/- 5.0% |
| 72 | HBoV4-NI NS1 | Virus | 62.2% | 51.4% | 56.8% +/- 7.6% |
| 73 | HHV6B AVP | Virus | 74.0% | 62.4% | 68.2% +/- 8.2% |
| 74 | HHV6A pp100 | Virus | 25.5% | 14.0% | 19.7% +/- 8.1% |
| 75 | HHV7 MTP | Virus | 89.0% | 90.2% | 89.6% +/- 0.9% |
| 76 | HAdV-D pIIIa | Virus | 95.8% | 96.4% | 96.1% +/- 0.4% |
| 77 | HAdV-A SSB | Virus | 72.8% | 61.9% | 67.4% +/- 7.7% |
| 78 | HAdV-B pIIIa | Virus | 95.3% | 95.5% | 95.4% +/- 0.2% |
| 79 | HIV-1MB Pro | Virus | 5.1% | 3.2% | 4.1% +/- 1.3% |
| 80 | RSV NP | Virus | 100.0% | 100.0% | 100.0% +/- 0.0% |
| 81 | HPeV-1 | Virus | 34.1% | 16.8% | 25.4% +/- 12.2% |
| 82 | HPV34 LP | Virus | 2.5% | 0.8% | 1.7% +/- 1.2% |
| 83 | HPV9 LP | Virus | 16.0% | 6.2% | 11.1% +/- 6.9% |
| 84 | HPeV-6 PolyP | Virus | 89.0% | 82.5% | 85.7% +/- 4.6% |
| 85 | HPIV2 NC | Virus | 95.6% | 89.6% | 92.6% +/- 4.2% |
| 86 | HRV3 NC | Virus | 100.0% | 100.0% | 100.0% +/- 0.0% |
| 87 | HRV-1A Pro | Virus | 99.9% | 99.9% | 99.9% +/- 0.0% |
| 88 | HIV-2A VPR | Virus | 12.6% | 5.7% | 9.2% +/- 4.9% |
| 89 | IBV M1 | Virus | 35.2% | 11.2% | 23.2% +/- 17.0% |
| 90 | ICV NP | Virus | 95.2% | 90.0% | 92.6% +/- 3.7% |
| 91 | NoV-GII VP1 | Virus | 98.4% | 98.4% | 98.4% +/- 0.0% |
| 92 | NoV-GI NSP | Virus | 94.5% | 95.4% | 95.0% +/- 0.6% |
| 93 | SaV PolyP | Virus | 19.6% | 19.9% | 19.7% +/- 0.2% |
| 94 | TTMDV1 | Virus | 44.0% | 34.5% | 39.2% +/- 6.7% |
| 95 | TTV7 pORF2 | Virus | 6.3% | 1.0% | 3.7% +/- 3.8% |
| 96 | VSIV NS | Virus | 18.0% | 3.6% | 10.8% +/- 10.2% |
| 97 | HCoV-HKU1 NC | Virus | 80.2% | 66.0% | 73.1% +/- 10.0% |
| 98 | HCoV-OC43 NC | Virus | 98.1% | 96.0% | 97.1% +/- 1.5% |
| 99 | HCoV-229E NC | Virus | 96.9% | 95.8% | 96.3% +/- 0.8% |
| 100 | HCoV-NL63 NC | Virus | 100.0% | 99.8% | 99.9% +/- 0.2% |
| 101 | RUBV E2 | Virus | 21.2% | 11.1% | 16.2% +/- 7.2% |
| 102 | TTMDV1 gp1 | Virus | 69.3% | 70.2% | 69.8% +/- 0.6% |
| 103 | SFV NSP3 | Virus | 3.7% | 3.1% | 3.4% +/- 0.4% |
| 104 | SINV NSP3 | Virus | 1.2% | 0.8% | 1.0% +/- 0.3% |
| 105 | TiV P | Virus | 5.2% | 4.6% | 4.9% +/- 0.4% |
| 106 | YF-VAX M | Virus | 2.4% | 1.4% | 1.9% +/- 0.7% |
| 107 | EBV BFRF3 | Virus | 73.5% | 79.5% | 76.5% +/- 4.2% |
| 108 | HPV5 E7 | Virus | 6.0% | 4.1% | 5.1% +/- 1.3% |
| 109 | HPV6 E6 | Virus | 0.8% | 0.8% | 0.8% +/- 0.0% |
| 110 | HPV8 E7 | Virus | 1.7% | 2.3% | 2.0% +/- 0.4% |
| 111 | HPV16 E7 | Virus | 3.4% | 4.9% | 4.1% +/- 1.0% |
| 112 | IAV M1 | Virus | 79.2% | 68.0% | 73.6% +/- 7.9% |
| 113 | SV40 small t | Virus | 11.7% | 6.5% | 9.1% +/- 3.7% |
| 114 | CVB4 VP1 | Virus | 82.4% | 82.5% | 82.4% +/- 0.1% |
| 115 | CHIKV NSP3 | Virus | 2.1% | 2.5% | 2.3% +/- 0.3% |
| 116 | EV-C VP1 | Virus | 70.0% | 66.8% | 68.4% +/- 2.3% |
| 117 | HHV1 US4 | Virus | 34.2% | 45.6% | 39.9% +/- 8.1% |
| 118 | HBV Pol | Virus | 5.3% | 2.8% | 4.1% +/- 1.7% |
| 119 | HERV Evn | Virus | 2.1% | 1.1% | 1.6% +/- 0.7% |
| 120 | HCMV UL32 | Virus | 49.1% | 48.9% | 49.0% +/- 0.1% |
| 121 | HAdV-C pIII | Virus | 95.4% | 96.8% | 96.1% +/- 1.0% |
| 122 | HMPV NP | Virus | 100.0% | 100.0% | 100.0% +/- 0.0% |
| 123 | HPV6b L1 | Virus | 70.8% | 52.4% | 61.6% +/- 13.0% |
| 124 | HPV11 E1 | Virus | 2.2% | 1.5% | 1.8% +/- 0.5% |
| 125 | HPV31 E7 | Virus | 10.2% | 7.3% | 8.7% +/- 2.1% |
| 126 | HPV33 L1 | Virus | 49.2% | 32.4% | 40.8% +/- 11.9% |
| 127 | HPV35 L1 | Virus | 62.0% | 32.1% | 47.0% +/- 21.2% |
| 128 | HPV39 L2 | Virus | 2.2% | 0.7% | 1.4% +/- 1.1% |
| 129 | HPV45 L2 | Virus | 2.4% | 0.4% | 1.4% +/- 1.4% |
| 130 | HPV51 L1 | Virus | 43.0% | 24.9% | 34.0% +/- 12.8% |
| 131 | HPV58 L2 | Virus | 2.5% | 0.6% | 1.5% +/- 1.3% |
| 132 | RSV-B NC | Virus | 100.0% | 100.0% | 100.0% +/- 0.0% |
| 133 | MeV-VAX N | Virus | 97.6% | 94.2% | 95.9% +/- 2.4% |
| 134 | MuV-IA NP | Virus | 99.0% | 98.0% | 98.5% +/- 0.7% |
| 135 | MuV P | Virus | 22.7% | 22.3% | 22.5% +/- 0.3% |
| 136 | HBoV1 VP2 | Virus | 18.7% | 22.7% | 20.7% +/- 2.8% |
| 137 | EBV EBNA1 | Virus | 76.9% | 81.2% | 79.1% +/- 3.1% |
| 138 | SARS-CoV-2 NC | Virus | 30.5% | 47.0% | 38.7% +/- 11.7% |
| 139 | Control GFP | Control | 15.5% | 17.9% | 16.7% +/- 1.7% |
| 140 | IFN-α2 | Human | 2.1% | 2.5% | 2.3% +/- 0.3% |
| 141 | IFN-α4 | Human | 6.5% | 7.5% | 7.0% +/- 0.7% |
| 142 | p53 | Human | 3.5% | 4.5% | 4.0% +/- 0.7% |
| 143 | SARS-CoV-2 wRBD | Virus | 96.3% | 99.1% | 97.7% +/- 1.9% |
| 144 | SARS-CoV-2 oRBD | Virus | 10.4% | 10.8% | 10.6% +/- 0.3% |
| 145 | HCoV-229E RBD | Virus | 99.2% | 99.4% | 99.3% +/- 0.1% |
| 146 | HCoV-OC43 RBD | Virus | 100.0% | 99.9% | 99.9% +/- 0.1% |
| 147 | HCoV-HKU1 RBD | Virus | 96.4% | 97.6% | 97.0% +/- 0.8% |
